# Supplementary material for: A giant virus infecting the amoeboflagellate Naegleria
Source: Nat Commun. 2024 Apr 24;15:3307. doi: 10.1038/s41467-024-47308-2 (PMC11043551; doi:10.1038/s41467-024-47308-2)
Supplement: Supplementary file 1 — Supplementary Information [file 41467_2024_47308_MOESM1_ESM.pdf]

Supplementary Information

A giant virus infecting the amoeboflagellate *Naegleria*

Patrick Arthofer, Florian Panhölzl, Vincent Delafont, Alban Hay, Siegfried Reipert, Norbert Cyran, Stefanie Wienkoop, Anouk Willemsen, Ines Sifaoui, Iñigo Arberas-Jiménez, Frederik Schulz, Jacob Lorenzo-Morales, Matthias Horn

Content

- **Supplementary Figure 1**: Phenotypic effect of Naegleriavirus on *Naegleria clarki* and *Naegleria fowleri*.
- **Supplementary Figure 2**: *Naegleria clarki* phenotypes of amoeba cells devoid of viral factories.
- **Supplementary Figure 3**: The effect of Naegleriavirus replication on amoeba hosts at different temperatures.
- **Supplementary Table 1**: Naegleriavirus genes potentially involved in metabolism, vesicle trafficking, and genes encoding for intein-containing proteins and transposases.
- **Supplementary Table 2**: Naegleriavirus genes with sequence similarity to eukaryotic genes.
- **Supplementary Table 3**: Primers and probes.

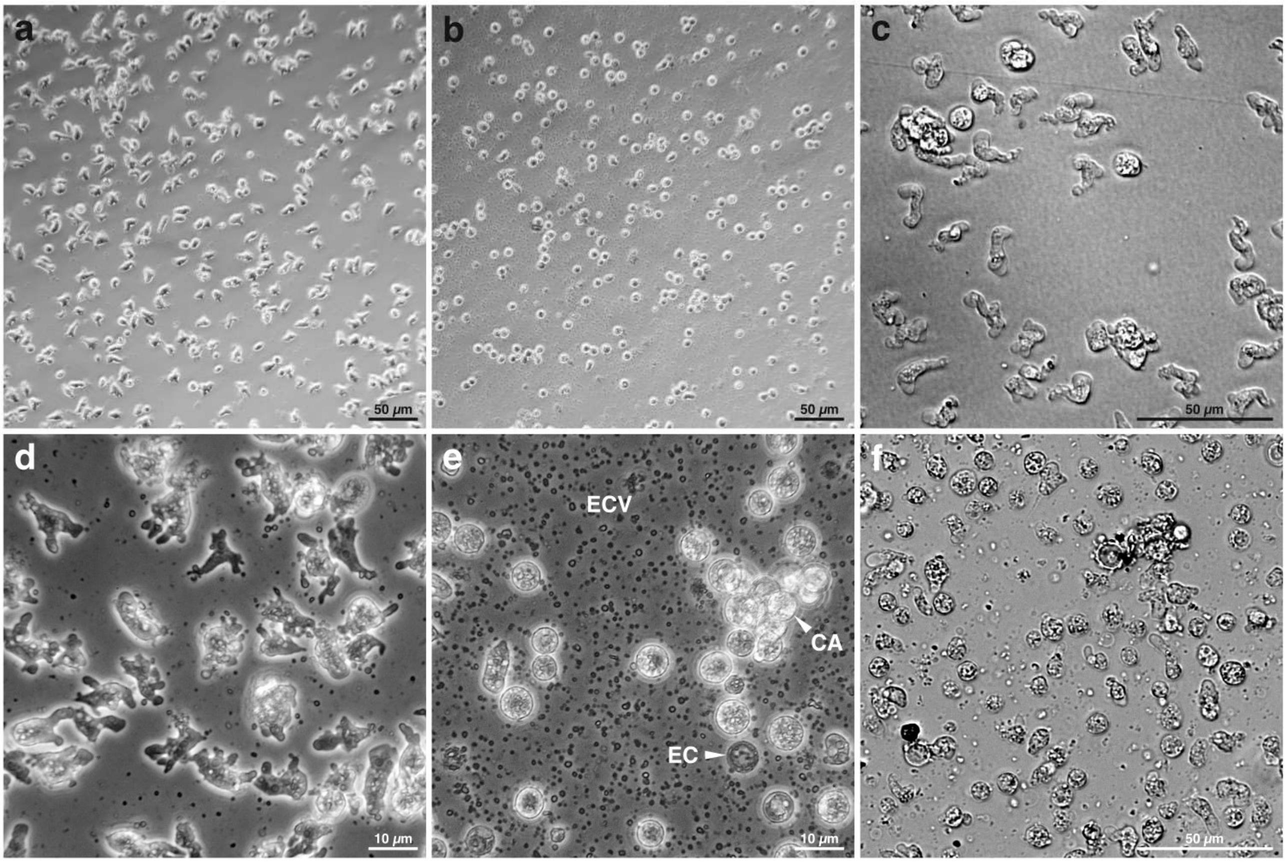

Supplementary Figure 1: Phenotypic effect of *Naegleriavirus* on *Naegleria clarki* and *Naegleria fowleri*. **(a, d)** Uninfected *N. clarki* cells. **(b, e)** The same *N. clarki* culture 24 hours post NiV infection. **(c)** Uninfected *N. fowleri* cells (72 hours post experimental start) **(f)** *N. fowleri* 72 hours post NiV infection. The experiments were carried out with an MOI of 10. EC=cyst devoid of cellular content. ECV=extracellular vesicles. CA=cyst aggregates.

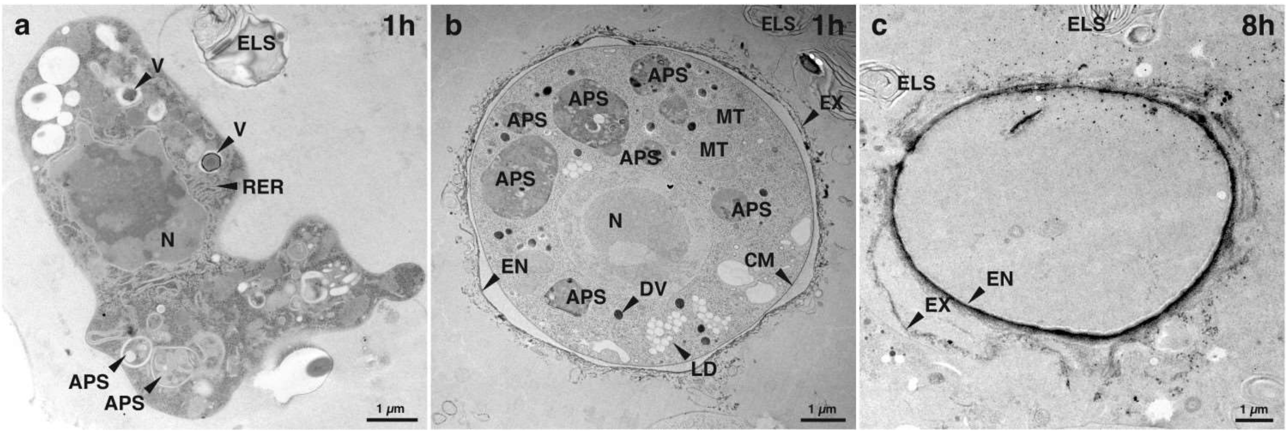

Supplementary Figure 2: ***Naegleria clarki*** **phenotypes of amoeba cells devoid of viral factories.** Numbers indicate hours post infection (hpi). **(a)** Infected amoeba displaying the nucleus including heavily condensed heterochromatin, the rough endoplasmic reticulum, and large autophagosomes. **(b)** Rounded cell undergoing encystation forming an endocyst surrounded by an exocyst wall. Autophagosomes and lipid droplets can be seen. **(c)** Empty cyst surrounded by extracellular multilamellar structures also visible in (a) and (b). Extracellular multi-lamellar structures could either be produced actively by the trophozoites or alternatively result from cell lysis. APS=autophagosome. CM=cell membrane. ELS=extracellular multi-lamellar structure. EN=endocyst wall. EX=exocyst wall. MT=mitochondrion. N=nucleus. V= virion. DV= dark vesicles. LD=lipid droplets. RER=rough endoplasmatic reticulum. All images were produced from cryofixed samples.

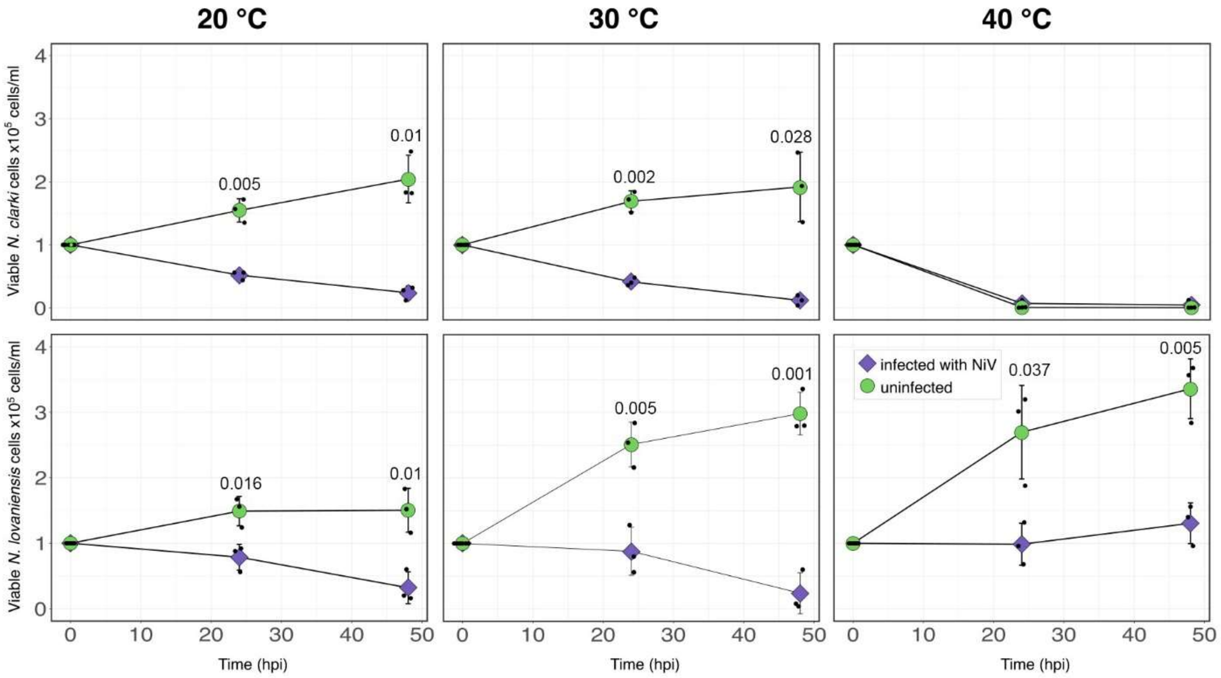

Supplementary Figure 3. The effect of Naegleriavirus replication on amoeba hosts at different temperatures. Amoeba cell numbers for *N. clarki* (upper panels) and *N. lovaniensis* (lower panels) were recorded for 48h with or without Naegleriavirus at incubation temperatures of 20, 30, and 40 °C. MOI=1. A (two-tailed) Welch's t-test was used for statistical analysis; n = 3 independent replicates; p-values are depicted (rounded to the third decimal). Colored circles indicate the mean, whiskers indicate the standard deviation.

Supplementary Table 1: Naegleriavirus genes potentially involved in metabolism, vesicle trafficking, and genes encoding for intein-containing proteins and transposases.

CDS / locus tag	Description
**Metabolism**	
IMIMIMCATVN1_00086	beta-Oxidation
IMIMIMCATVN1_00832	acyl-CoA synthesis AMP-binding enzyme
IMIMIMCATVN1_00840	ureohydrolase (polyamine biosynthesis)
IMIMIMCATVN1_00880	ornithine decarboxylase
IMIMIMCATVN1_00264	capsular polysaccharide biosynthesis protein Cap F
IMIMIMCATVN1_00302	putative dTDP-d-glucose 4 6-dehydratase
IMIMIMCATVN1_00303	UDP-N-acetylglucosamine 2-epimerase WecB-like protein
**Vesicle trafficking**	
IMIMIMCATVN1_00962	Vesicle fusing ATPase
IMIMIMCATVN1_0076	Target SNARE-coiled-coil homology domain-containing proteins
IMIMIMCATVN1_00229	Target SNARE-coiled-coil homology domain-containing proteins
IMIMIMCATVN1_00014<br>IMIMIMCATVN1_00015<br>IMIMIMCATVN1_00373	Dynamin superfamily proteins
IMIMIMCATVN1_00237<br>IMIMIMCATVN1_00396<br>IMIMIMCATVN1_00417<br>IMIMIMCATVN1_00494<br>IMIMIMCATVN1_00531	Small GTPases (Rab subfamily)
**Intein-containing proteins**	
IMIMIMCATVN1_00600	helicase, superfamily 3, DNA virus/DNA primase
IMIMIMCATVN1_00613	phage/plasmid domain-containing protein, DNA-directed RNA polymerase subunit 2
IMIMIMCATVN1_00632	DNA-directed RNA polymerase subunit 1
IMIMIMCATVN1_00635	replication factor C subunit 5
IMIMIMCATVN1_00735	P-loop containing nucleoside triphosphate hydrolase homologous superfamily type protein
IMIMIMCATVN1_00783	replication factor C subunit 4
IMIMIMCATVN1_00801	P-loop containing nucleoside triphosphate hydrolase homologous superfamily type protein

Transposases	
IMIMIMCATVN1_00913	putative transposase, IS891/IS1136/IS1341 domain/Transposase IS605, OrfB, C-terminal domain-containing protein
IMIMIMCATVN1_00081<br>IMIMIMCATVN1_00114<br>IMIMIMCATVN1_00337<br>IMIMIMCATVN1_00913<br>IMIMIMCATVN1_00954	Transposase IS605, OrfB, C-terminal domain-containing proteins
IMIMIMCATVN1_00234	putative transposase, helix-turn-helix domain-containing protein
IMIMIMCATVN1_00287	Transposase

Supplementary Table 2: Naegleriavirus genes with sequence similarity to eukaryotic genes.

CDS / locus tag	Description
**Sister branch to or within *Naegleria* homologs**	
IMIMIMCATVN1_00038	BCS1, N-terminal domain/P-loop containing nucleoside triphosphatehydrolase homologous superfamily domain-containing protein
IMIMIMCATVN1_00074	Bax inhibitor 1-related family protein
IMIMIMCATVN1_00076	Target SNARE coiled-coil homology domain-containing protein
IMIMIMCATVN1_00080	Mitochondrial chaperone BCS1
IMIMIMCATVN1_00229	Target SNARE coiled-coil homology domain containing protein
IMIMIMCATVN1_00346	Regulator of chromosome condensation 1/beta-lactamase-inhibitor protein II homologous superfamily domain-containing protein
IMIMIMCATVN1_00426	Heat shock protein 70 family
IMIMIMCATVN1_00493	DNA helicase, UvrD/REP type
IMIMIMCATVN1_00735	Ubiquitin-like superfamily domain-containing protein
IMIMIMCATVN1_00958	Regulator of chromosome condensation 1/beta-lactamase-inhibitor homologous superfamily protein II
IMIMIMCATVN1_00962	Vesicle-fusing ATPase
**Affiliated with eukaryotes other than *Naegleria***	
IMIMIMCATVN1_00172	RGS domain and START-like domain-containing protein
IMIMIMCATVN1_00202	Kelch-type beta propeller homologous superfamilydomain-containing protein
IMIMIMCATVN1_00227	von Willebrand factor A-like homologous superfamily domain-containing protein
IMIMIMCATVN1_00237	Small GTPase
IMIMIMCATVN1_00253	Synaptobrevin
IMIMIMCATVN1_00283	Methionine--tRNA ligase
IMIMIMCATVN1_00317	Kelch-type beta propeller homologous superfamilydomain-containing protein
IMIMIMCATVN1_00396	Small GTPase
IMIMIMCATVN1_00494	Small GTPase
IMIMIMCATVN1_00517	Thioredoxin-like superfamily protein
IMIMIMCATVN1_00519	Ubiquitin-conjugating enzyme/RWD-like superfamilyprotein
IMIMIMCATVN1_00528	Ubiquitin-conjugating enzyme/RWD-like superfamilydomain-containing protein
IMIMIMCATVN1_00577	Peptide chain release factor eRF1/aRF1
IMIMIMCATVN1_00635	Intein-containing replication factor C subunit 5

IMIMIMCATVN1_00665	S-phase kinase-associated protein 1
IMIMIMCATVN1_00681	Ubiquitin-conjugating enzyme/RWD-like protein
IMIMIMCATVN1_00734	Heat shock protein 70
IMIMIMCATVN1_00845	DEAD/DEAH box helicase domain/DEAD-box type, Q motif/Helicase,C-terminal/Helicase superfamily 1/2, ATP-binding domain-containing protein
IMIMIMCATVN1_00938	PH-like superfamily/PAS superfamily/ RGS superfamily domain-containing protein
**Hypothetical proteins**	
IMIMIMCATVN1_00102<br>IMIMIMCATVN1_00169<br>IMIMIMCATVN1_00178<br>IMIMIMCATVN1_00270<br>IMIMIMCATVN1_00277<br>IMIMIMCATVN1_00588<br>IMIMIMCATVN1_00978	hypothetical protein

Supplementary Table 3: Primers and probes.

Name	Sequence	Reference	Formamide concentration / Annealing Temperature
**FISH**			
Euk516	5’-GGAGGGCAAGTCTGGT-3’	(1)	25%
Nag1088	5’-GTGGCCCACGACAGCTTT-3’	(4), (2)	25%
Eub338-1-3	5’-GCTGCCTCCCGTAGGAGT-3’<br>5’-GCACCCACCCGTAGGTGT-3’<br>5’-GCTCCCACCCGTAGGTGT-3’	(1), (3)	25%
**PCR**			
DNA_pol_NiV_1704F	5'-TCATCTTGCTCCCGTTTTAGA -3'	this study	60 °C
DNA_pol_NiV_1880R	5'- ACAGTTGCTCCTTCATAACCA -3'	this study	60 °C
DNA_pol_NiV_1512F	5'-AGATAGAGCAATCATCGCAAAGT-3'	this study	57 °C
DNA_pol_NiV_2352R	5'-TTCTTGTAATACCTCCGCCATT-3'	this study	57 °C

1. Amann, R. I., Krumholz, L. & Stahl, D. A. Fluorescent-oligonucleotide probing of whole cells for determinative, phylogenetic, and environmental studies in microbiology. J. Bacteriol. 172, 762–770 (1990).
2. Arthofer, P., Delafont, V., Willemsen, A., Panhölzl, F. & Horn, M. Defensive symbiosis against giant viruses in amoebae. Proc. Natl. Acad. Sci. U. S. A. 119, e2205856119 (2022).
3. Daims, H., Brühl, A., Amann, R., Schleifer, K. H. & Wagner, M. The domain-specific probe EUB338 is insufficient for the detection of all Bacteria: development and evaluation of a more comprehensive probe set. Syst. Appl. Microbiol. 22, 434–444 (1999).
4. Grimm, D. et al. Development of 18S rRNA-targeted oligonucleotide probes for specific detection of *Hartmannella* and *Naegleria* in *Legionella*-positive environmental samples. Syst. Appl. Microbiol. 24, 76–82 (2001).
