# Supplementary material for: A giant virus infecting the amoeboflagellate Naegleria
Source: Nat Commun. 2024 Apr 24;15:3307. doi: 10.1038/s41467-024-47308-2 (PMC11043551; doi:10.1038/s41467-024-47308-2)
Supplement: Supplementary file 3 — Description of Additional Supplementary Files [file 41467_2024_47308_MOESM3_ESM.pdf]

Supplementary Data

A giant virus infecting the amoeboflagellate *Naegleria*

Patrick Arthofer, Florian Panhölzl, Vincent Delafont, Alban Hay, Siegfried Reipert, Norbert Cyran, Stefanie Wienkoop, Anouk Willemsen, Ines Sifaoui, Iñigo Arberas-Jiménez, Frederik Schulz, Jacob Lorenzo-Morales, Matthias Horn

Title: Supplementary data set 1
Description: The NiV genome. Manually curated annotation of NiV genes, best blast hits, mapping to EggNOG and KEGG. *[Provided online as separate spreadsheet]*

Title: Supplementary data set 2
Description: The relationship of Naegleriavirus with other Nucleocitoviricota. Maximum likelihood tree constructed with three Nucleocitoviricota core genes, DNA polymerase family B, A18-like helicase, and poxvirus late transcription factor VLTF3. File in Newick format. *[Provided online as separate text file]*

Title: Supplementary data set 3
Description: The NiV virion proteome. List of proteins detected in purified NiV particles. *[Provided online as separate spreadsheet]*
