# Supplementary material for: A giant virus infecting the amoeboflagellate Naegleria
Source: Nat Commun. 2024 Apr 24;15:3307. doi: 10.1038/s41467-024-47308-2 (PMC11043551; doi:10.1038/s41467-024-47308-2)
Supplement: Supplementary file 7 — Reporting Summary [file 41467_2024_47308_MOESM7_ESM.pdf]

nature portfolio

Corresponding author(s): Matthias Horn

Last updated by author(s): Mar 13, 2024

Reporting Summary

Nature Portfolio wishes to improve the reproducibility of the work that we publish. This form provides structure for consistency and transparency in reporting. For further information on Nature Portfolio policies, see our Editorial Policies and the Editorial Policy Checklist.

Statistics

For all statistical analyses, confirm that the following items are present in the figure legend, table legend, main text, or Methods section.

n/a	Confirmed	
☐	☒	The exact sample size (*n*) for each experimental group/condition, given as a discrete number and unit of measurement
☒	☐	A statement on whether measurements were taken from distinct samples or whether the same sample was measured repeatedly
☐	☒	The statistical test(s) used AND whether they are one- or two-sided<br>*Only common tests should be described solely by name; describe more complex techniques in the Methods section.*
☒	☐	A description of all covariates tested
☒	☐	A description of any assumptions or corrections, such as tests of normality and adjustment for multiple comparisons
☐	☒	A full description of the statistical parameters including central tendency (e.g. means) or other basic estimates (e.g. regression coefficient) AND variation (e.g. standard deviation) or associated estimates of uncertainty (e.g. confidence intervals)
☐	☒	For null hypothesis testing, the test statistic (e.g. *F*, *t*, *r*) with confidence intervals, effect sizes, degrees of freedom and *P* value noted<br>*Give P values as exact values whenever suitable.*
☒	☐	For Bayesian analysis, information on the choice of priors and Markov chain Monte Carlo settings
☒	☐	For hierarchical and complex designs, identification of the appropriate level for tests and full reporting of outcomes
☒	☐	Estimates of effect sizes (e.g. Cohen's *d*, Pearson's *r*), indicating how they were calculated

Our web collection on statistics for biologists contains articles on many of the points above.

Software and code

Policy information about availability of computer code

Data collection	No specific software was used for data collection.
Data analysis	MaxQuant v1.6.5.0; FastQC v.11.9; FASTX-Toolkit v0.0.13; prinseq-lite.pl v0.20.4; canu v2; Unicycler v0.4.8; PROKKA v1.14.6; tRNAscan-SE v2.0.9; Unipro Ugene v37.1; InterProScan v5.45-80.0; BLASTP, v2.10.0; eggNOG-mapper v2; MAFFT v7.520; trimAl v1.41; SeaView v5.05; IQ-TREE 2 v2.2.7; iTOL v6.7.6; Seaview software v4.7; MUSCLE v3.8.31; cdhit v4.8.1; MAFFT v7.520; trimal v1.4; fast tree v2.1.11; R-studio v4.2.1

For manuscripts utilizing custom algorithms or software that are central to the research but not yet described in published literature, software must be made available to editors and reviewers. We strongly encourage code deposition in a community repository (e.g. GitHub). See the Nature Portfolio guidelines for submitting code & software for further information.

Data

Policy information about availability of data

All manuscripts must include a data availability statement. This statement should provide the following information, where applicable:

- Accession codes, unique identifiers, or web links for publicly available datasets
- A description of any restrictions on data availability
- For clinical datasets or third party data, please ensure that the statement adheres to our policy

Sequencing data and the annotated Naegleriavirus genome generated in this study has been deposited in Genbank/ENA/DDBJ under accession code OZ003748 [https://www.ncbi.nlm.nih.gov/nuccore/OZ003748.1/] and as BioProject PRJEB72011 [https://www.ncbi.nlm.nih.gov/bioproject/1069539]. Proteome data has been

deposited at ProteomeXchange under accession code PXD044853 [http://proteomecentral.proteomexchange.org/cgi/GetDataset?ID=PXD044853]. Supplementary phylogenetic tree files are available at figshare under the DOI 10.6084/m9.figshare.25117901 [https://doi.org/10.6084/m9.figshare.25117901]. Source data for Figure 8 and Supplementary Fig. 3 are provided with this paper.

Research involving human participants, their data, or biological material

Policy information about studies with human participants or human data. See also policy information about sex, gender (identity/presentation), and sexual orientation and race, ethnicity and racism.

Reporting on sex and gender	n/a
Reporting on race, ethnicity, or other socially relevant groupings	n/a
Population characteristics	n/a
Recruitment	n/a
Ethics oversight	n/a

Note that full information on the approval of the study protocol must also be provided in the manuscript.

Field-specific reporting

Please select the one below that is the best fit for your research. If you are not sure, read the appropriate sections before making your selection.

[x] Life sciences [ ] Behavioural & social sciences [ ] Ecological, evolutionary & environmental sciences

For a reference copy of the document with all sections, see nature.com/documents/nr-reporting-summary-flat.pdf

Life sciences study design

All studies must disclose on these points even when the disclosure is negative.

Sample size	The study focuses on the characterization of a novel giant virus infecting the unicellular eukaryote Naegleria. For genomic, phylogenetic and phylogenomic analyses, the sizes of sequence data sets varied dependent on the analysis and available nucleotide or protein sequences. If not the entire data set of sequences could be included for computational reasons, the data set was de-replicated to include representative sequences.<br>For the wet-lab part of the study (growth and infection experiments) no statistical method was used to predetermine sample size. A sample size of n=3 (number of independent repeats) was chosen, which is sufficient for the statistical tests used.
Data exclusions	No data was excluded from this study.
Replication	All experiments were repeated (at least) three times. All reported experiments were repeatable.<br>All computational analyses can be replicated with the parameters and program versions provided in the methods section.
Randomization	Not applicable as the quantitative experimental approaches used in this study are not susceptible to potential biases.
Blinding	Not applicable as the quantitative methods used for data acquisition in this study are not susceptible to potential biases.

Reporting for specific materials, systems and methods

We require information from authors about some types of materials, experimental systems and methods used in many studies. Here, indicate whether each material, system or method listed is relevant to your study. If you are not sure if a list item applies to your research, read the appropriate section before selecting a response.

Materials & experimental systems

n/a	Involved in the study	
[x]	[ ]	Antibodies
[x]	[ ]	Eukaryotic cell lines
[x]	[ ]	Palaeontology and archaeology
[x]	[ ]	Animals and other organisms
[x]	[ ]	Clinical data
[x]	[ ]	Dual use research of concern
[x]	[ ]	Plants

Methods

n/a	Involved in the study	
[x]	[ ]	ChIP-seq
[x]	[ ]	Flow cytometry
[x]	[ ]	MRI-based neuroimaging
